# “RAF” neighborhood: Protein–protein interaction in the Raf/Mek/Erk pathway

**DOI:** 10.1016/j.febslet.2014.06.025

**Published:** 2014-08-01

**Authors:** Botond Cseh, Eszter Doma, Manuela Baccarini

**Affiliations:** Department of Microbiology, Immunobiology and Genetics, Center of Molecular Biology, Max F. Perutz Laboratories, University of Vienna, Doktor-Bohr-Gasse 9, 1030 Vienna, Austria

**Keywords:** Ras, Raf, Mek, Erk, Protein–protein interaction, Kinase inhibitor, Cancer therapy

## Abstract

The Raf/Mek/Erk signaling pathway, activated downstream of Ras primarily to promote proliferation, represents the best studied of the evolutionary conserved MAPK cascades. The investigation of the pathway has continued unabated since its discovery roughly 30 years ago. In the last decade, however, the identification of unexpected in vivo functions of pathway components, as well as the discovery of Raf mutations in human cancer, the ensuing quest for inhibitors, and the efforts to understand their mechanism of action, have boosted interest tremendously. From this large body of work, protein–protein interaction has emerged as a recurrent, crucial theme. This review focuses on the role of protein complexes in the regulation of the Raf/Mek/Erk pathway and in its cross-talk with other signaling cascades. Mapping these interactions and finding a way of exploiting them for therapeutic purposes is one of the challenges of future molecule-targeted therapy.

## 1 Introduction

**Fig. 1:**
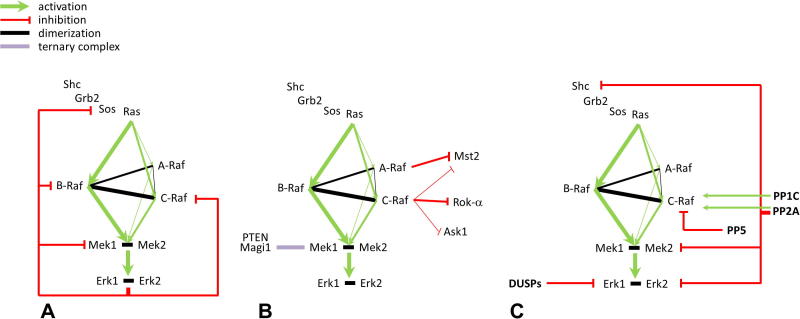
The Raf/Mek/Erk Pathway. (A) *Schematic wiring of the pathway* – the Raf/Mek/Erk pathway is a three-tiered kinase cascade that operates downstream of the small GTPase Ras. The three Rafs bind Ras with different affinities, which determine their sensitivity to activated Ras. Rafs, in particular B-Raf and C-Raf, form homo- and hetero-dimers which phosphorylate and activate Meks, which in turn transfer the signal to Erks. Erks have many substrates whose activation leads to a variety of biological responses. Knockout studies have revealed that B-Raf is essential for Mek/Erk activation downstream of Ras; A-Raf and C-Raf can also activate Erk upon heterodimerization with B-Raf. Raf and Mek1 are the recipients of negative feedback phosphorylation by Erk, which determines the strength and duration of the Erk signal. (B) *Cross-talk with other pathways* – A-Raf and C-Raf can transmit signals in a Mek-independent manner, by communicating with parallel pathways. Both of them bind to and inhibit the proapoptotic kinase Mst2. In addition, C-Raf can bind and inhibit another proapoptotic kinase, Ask1, and the cytoskeleton-based Rok-α. An intact C-Raf:Rok-α complex is required for cell shape and motility, it impacts on angiogenesis and it is essential for preventing differentiation in Ras-driven epidermal tumors. Similar to C-Raf, Mek1 impacts a parallel pathway leading to Akt phosphorylation, by preventing PTEN-Mediated PIP3 turnover in the context of a Mek1/Magi1/PTEN ternary complex. (C) *Phosphatases interacting with Erk pathway components* – phosphatases play a dual role in Erk pathway regulation: a positive role, by facilitating C-Raf activation (PP2A, PP1C; green arrows) and a negative role (red lines) by dephosphorylating Shc, Mek and Erk (PP2A), C-Raf (PP5) or Erk (DUSPs). In Fig. 1B, line thickness is proportional to the strength and significance of the interactions.

The Raf/Mek/Erk signal transduction pathway is the best studied of the four mitogen-activated protein kinase (MAPK) cascades present in vertebrates (Fig. 1). It is activated by growth factors, hormones and cytokines and has been shown to regulate proliferation but also differentiation, survival, senescence, and migration [1]. Typically, ligand-binding to a cell surface receptor induces a wave of tyrosine phosphorylation (autophosphorylation in the case of receptor tyrosine kinases, or phosphorylation by receptor-associated kinases if the receptor itself lacks catalytic activity) resulting in the generation of phosphotyrosine binding sites for adaptor proteins such as growth factor receptor-bound protein 2 (GRB2). GRB2 mediates the membrane translocation of the guanine nucleotide exchange factor (GEF) son of sevenless (SOS), which in turn activates the membrane bound GTPase Ras [1]. Ras functions as a binary molecular switch that cycles between inactive GDP-bound and active GTP-bound states with the help of GEFs and GTPase activating proteins (GAPs). The exchange of GTP for GDP by SOS changes the conformation of Ras, allowing its interaction with effectors such as Raf [2]. GTP-bound Ras recruits Raf to the plasma membrane and enables it to phosphorylate its only substrates, Mek1 and Mek2 [3]. These dual specificity kinases mediate the activation of Erk1 and Erk2, enabling them to phosphorylate a variety of nuclear and cytoplasmic targets [4].

Mammals express three Raf isoforms, A-, B-, and C-Raf (the latter also called Raf-1) with distinct affinities for both the activator, Ras, and the downstream target Mek. B-Raf is the isoform most similar to Rafs expressed in lower organisms [5], and can therefore be considered the archetypal mammalian Mek kinase. A-Raf and C-Raf have evolved to fulfill other, potentially Mek-independent requirements [6,7]. Accordingly, growth factor-stimulated Erk activation is decreased in B-Raf-, but not A-Raf or C-Raf -deficient cells [8–12]. Similarly, the high occurrence of B-Raf but not A-Raf or C-Raf mutations in human cancers implies a dominant role for B-Raf in signaling to the Erk pathway [13,14].

## 2 Homo and heterodimers in Raf activation

Homo- and heterodimerization play an important role in the Erk pathway, whether by allowing the propagation of the signal to downstream effectors, by orchestrating feedback loops within the pathway, or by enabling communication with parallel signaling circuits [15]. Dimerization of pathway components can result in their activation (Raf) or inhibition (Mek). Furthermore, binding to different scaffolds can influence the localization of the components to different cellular compartments, increasing signal fidelity and strength [16]. One of these scaffolds, the pseudokinase Ksr, interacts with Raf, Mek and Erk and localizes to the plasma membrane in a Ras-dependent manner [17].

Activation of Raf occurs via a complex, yet incompletely understood mechanism requiring membrane translocation, regulatory phosphorylation/dephosphorylation events [16] and, crucially, allosteric activation in the context of a side-to-side dimer comprising two Raf molecules or a Raf and a Ksr molecule [18–22]. Raf:Raf and Raf:Ksr dimerization depends on the dimer interface, a region located in the kinase domain, and in particular on a cluster of basic residues comprising B-RafR509, C-RafR401 and KsrR615 [21]. When these critical arginine residues are mutated to histidine (B-RafR509H, C-RafR401H, KsrR615H), activation does not take place. Conversely, the B-RafE586K mutation, which enhances dimerization and possibly allosteric transactivation, increases Erk signaling [23]. Growth factor-induced Raf dimerization can also be inhibited by an 18 amino acid peptide able to bind C-Raf and B-Raf, resulting in decreased Mek activation [23].

**Fig. 2:**
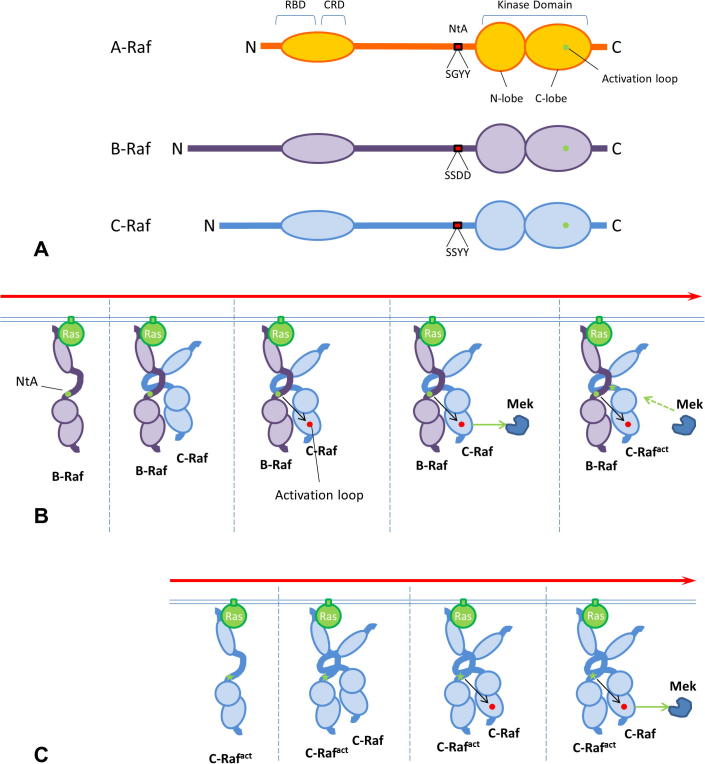
A Model of Raf transactivation. (A) *Conserved domains –* all Rafs share N-terminal Ras-binding domains (RBD) and cysteine-rich domains (CRD), both required for membrane recruitment. The Kinase Domain is located in the C-terminus, the activation loop is highlighted in green. Upstream of the Kinase Domain, the N-terminal Acidic motif (NtA; red boxes) contains phosphorylatable tyrosine residues (YY301/2 in A-Raf, Y340/41 in C-Raf), whereas B-Raf features aspartates in the corresponding region (D448/9). One Serine residue is conserved in all Raf proteins (S299 in A-Raf, S338 in C-Raf and S445 in B-Raf), but is constitutively phosphorylated only in B-Raf. (B) *Raf transactivation* – activated Ras recruits B-Raf to the plasma membrane. Ras binding allows a further Raf monomer to bind and dimerize. In the dimer interface, the constitutively phosphorylated NtA of B-Raf (green dot) induces a conformational change that allows the cis-phosphorylation of the receiver kinase (here C-Raf), enabling it to phosphorylate Mek. Mek, in turn induces the phosphorylation of S338 in the C-Raf NtA, converting it to a transactivator. (C) As a transactivator, C-Raf can dissociate from B-Raf and dimerize with, and transactivate, further Raf molecules. This cycle results in signal amplification.

Of the three Raf kinases, only B-Raf is able to function as an allosteric activator in the context of the Raf heterodimers, a role independent of B-Raf kinase activity [14,19,24]. The molecular basis for this has recently been elucidated by the Shaw lab [22], who has shown that the ability of acting as an activator depends on the presence of negative charges in the Raf N-terminal acidic motif. In B-Raf, this motif is negatively charged due to the constitutive phosphorylation of Ser446 and/or 447, and to the presence of two aspartates at position 448/9 [25] (Fig. 2A). Allosteric activation by B-Raf induces cis-autophosporylation in the activation loop of the receiver kinase, i.e. C-Raf, and renders it able to phosphorylate Mek. Mek, in turn, phosphorylates the N-terminal acidic motif in C-Raf, converting it to an allosteric activator of other Rafs [22,26] (Fig. 2B and C). This model explains why C-Raf mutants devoid of kinase activity cannot function as activators, and why B-Raf can activate Mek directly as a homodimer [23]. Phosphorylated Ksr can also function as a transactivator; however, since Raf binding to Ksr induces limited kinase activity [27], in quiescent cells the constitutive association of Ksr with B-Raf may serve to prevent C-Raf binding to B-Raf, safeguarding against undue activation of the pathway [28].

Some naturally occurring mutants of B-Raf can bypass the requirement for dimerization-mediated activation. These mutations (i.e. G469A and V600E, 599insT) disrupt the interaction between the P-loop and activation-loop [14], resulting in a constitutively active B-Raf kinase largely resistant to the disruption of the dimer interface [29]. B-Raf V600E mutants require homodimerization for activation only when their binding to Ras is impaired [30]. Oncogenic Ras has also been shown to promote the binding of B-RafV600E-to wild-type C-Raf, which results in a weakening of V600E activity and of Erk activation [31]. This work implies that the B-RafV600E mutant is unable to transactivate C-Raf, which may explain why oncogenic Ras mutations and B-RafV600E appear to be mutually exclusive.

## 3 Signaling through Mek-Erk activation, negative feedback and pathway cross-talk

Although loss of function or conventional knockout studies have revealed distinct roles for all three kinases, embryonic- (B-Raf and C-Raf) [9,32] or post natal lethality (A-Raf) [33] has hindered the analysis of the role of Rafs in vivo. Conditional knockout models have provided an opportunity to bypass this difficulty, and to test the role of specific isoforms in the activation of the ERK pathway in different organs. In good agreement with the pivotal role of B-Raf in Raf activation, B-Raf has been identified as the essential Mek/Erk activator in the placenta during vascular development [34] and in oligodendrocyte differentiation and myelination [35]. In the context of cancer, keratinocyte-restricted B-Raf deletion reduces Ras-driven carcinogenesis, which is consistent with its importance in this type of skin tumors [36]. B-Raf is also the main Mek/Erk-activator in a RIP1Tag2 tumor model in which the Erk pathway is not mutationally activated. In this model, the B-Raf/Mek/Erk axis was proven to be the key determinant of the communication between tumor and microenvironment, promoting the secretion of proangiogenic factors [37].

Rafs transmit the signal downstream by phosphorylating Mek1 and Mek2 on Ser218/Ser222 and Ser222/Ser226, respectively. Mek1 and Mek2 form homo- and heterodimers in vitro and in vivo [38,39], but unlike Raf heterodimers, the Mek1-Mek2 heterodimer is stable and its amount does not depend on growth factor stimulation [39]. Although they share common targets, Mek1 and Mek2 have unique biological properties. While disruption of Mek1 in mice is recessive embryonic lethal [40], the Mek2 knockout mice are viable, fertile and have no apparent abnormalities [41]. This predicts that on the systemic level, Mek1 homodimers can compensate for the loss of heterodimers and Mek2 homodimers, while Mek2 homodimers cannot. Mek1 and Mek2 mediate the phosphorylation of Erk1 and Erk2, which also form dimers [42,43]. Akin to the situation with Meks, only one of the isoforms, Erk2, is necessary during embryonic life, more specifically for trophoblast development [44]. In contrast, Erk1 knockout mice show only some minor defects in T-cell development, decreased adiposity and facilitated learning [45–47]. Both Erk1 and Erk2 are positive regulators of proliferation and are thought to be largely redundant in this context [48]. The dimerization of Erks does not influence their translocation to the nucleus, but is essential for signaling [43,49]. To reach specific cellular compartments, Erk rather interacts with scaffold proteins such as Ksr1, paxillin, IQGAP, Sef, MP1 or MORG [50]. These scaffolds contact the same hydrophobic site on Erk that is also needed for its substrate binding. Therefore, for their proper functioning, Erks have to form dimers to be able to simultaneously bind the scaffold and their substrates. Interfering with either Erk-dimerization or Erk binding to their scaffolds results in loss of proliferation and transformation [43].

In addition to mediating part of the biological response to Raf activation, Erk exerts negative feedback on the pathway through the phosphorylation of SOS [51,52], C-Raf [53–55], B-Raf [20,56,57] and Mek1 [39] (Fig. 1A). In this most proximal feedback, Erk phosphorylates a specific residue in Mek1, T292, which is absent in Mek2; disruption of the Mek1-Mek2 dimer, either by Mek1 deletion or by introducing a mutation in the dimer interface, eliminates this feed-back loop resulting in increased and prolonged Mek2 and Erk phosphorylation [39]. How the regulatory phosphorylation of T292 on Mek1 controls Mek2 activation still remains elusive.

Inactivating Mek by inhibition or silencing also enhances growth factor-induced Akt activation [58–60]. Intriguingly, phosphorylation of the same residue responsible for the negative feedback loop described above, T292, by Erk, enables Mek1 to form a ternary complex with the 100 kDa isoform of the scaffold protein Magi1 and the lipid phosphatase PTEN. The Mek1/Magi1/PTEN complex mediates the translocation of PTEN to the membrane, where it affects the amount of PIP3 and ultimately Akt activity (Fig. 1B). Thus, Mek1 phosphorylation by Erk limits the activation of both the Erk and the Akt pathway. In vivo, this chain of events leads to changes in peripheral self-tolerance and myeloproliferation in the Mek1-deficient mice [60].

## 4 Interacting phosphatases – a double-edged sword

The onset and duration of the Raf/Mek/Erk signal is regulated by protein serine/threonine phosphatases (PSPs) and by the dual-specificity phosphatase family (DUSPs/MKPs) (Fig. 1C). Specifically, computational modeling of the pathway has shown that kinases control signal amplitude and phosphatases both signal amplitude and duration [61,62].

PSPs dephosphorylate phosphoserine and phosphothreonine residues. One of the most abundantly expressed PSPs, protein phosphatase 2A (PP2A) [63], can regulate the Raf/Mek/Erk pathway both positively and negatively [64]. As a positive regulator, PP2A associates with C-Raf [65–69] and Ksr1 [68] and dephosphorylates negative regulatory sites on both proteins, allowing their recruitment to the membrane [66–69] and leading to Mek and Erk activation [65–68]. A similar role in C-Raf activation has been described for the catalytic subunit of PP1C, which associates with C-Raf in Ras- and growth factor-stimulated cells [70]. In addition to promoting C-Raf activation, PP2A is also able to dephosphorylate Erk-dependent sites on C-Raf [53]. Since the sites have been described alternatively as negative regulatory [53,71] or activating [72], the significance of these dephosphorylation events for Raf activation is unclear.

As a negative regulator of the pathway, PP2A can dephosphorylate the adapter protein Shc [73], required downstream of some tyrosine kinase receptors for the activation of the Raf/Mek/Erk module; and it can dephosphorylate Mek and Erk proteins [74–76], thus inhibiting the cascade directly. A further negative regulator of the cascade, at the level of C-Raf, is Protein phosphatase 5 (PP5), which associates with C-Raf via its N-terminal tetratricopeptide (TPR) domain in growth factor stimulated cells. This interaction leads to the activation of PP5 catalytic activity and to the selective dephosphorylation of the activating serine residue at position 338, terminating the signal [77].

While PSPs dephosphorylate the Raf-dependent phosphoserine site on Mek and the Mek-dependent phosphothreonine sites on Erk, DUSPs dephosphorylate both threonine and tyrosine residues of the MAP-Kinases Erk, p38 and Jnk [78]. The family consists of highly similar phosphatases with distinct substrate preference. These are dictated by the interaction of a modular binding domain in the N-terminus of the DUSPs, consisting of a kinase interaction motif (KIM) and of an additional stretch of positively charged residues flanked by hydrophobic amino acids, with a ‘common docking’ (CD) site on the MAPK. As in the case of PP5, substrate binding is required to stimulate the activity of DUSP1, DUSP2, DUSP6 and DUSP9. DUSPs also differ in their subcellular localization. While DUSP1, DUSP2, DUSP3, DUSP4, DUSP5 are localized in the nucleus and dephosphorylate Erk, p38 and Jnk, the Erk-selective DUSP6, DUSP7 and DUSP9 are localized in the cytoplasm, and at least in the case of DUSP6, anchor inactive Erk in the cytosol [79] and transport dephosphorylated Erk from the nucleus back to the cytosol [80]. Thus, phosphatases serve multiple functions in the Raf/Mek/Erk pathway.

## 5 Mek-independent Raf-signaling

In addition to activating Mek downstream of B-Raf, C-Raf can communicate with other parallel pathways (Fig. 1B). C-Raf is able to bind and inhibit the proapoptotic proteins Ask-1 [81] and Mst-2 [82,83], counteracting apoptosis; however, C-Raf’s best defined interaction partner outside the Mek/Erk pathway is the Rok-α kinase, which controls cytoskeletal dynamics downstream of the small GTPase Rho [84]. The C-Raf:Rok-α interaction has various consequences, depending on the cell type in which it takes place. It regulates the migration of keratinocytes and fibroblasts in culture, and wound healing in vivo [85]. In Ras-induced skin carcinogenesis, C-Raf-mediated inhibition of Rok-α blocks keratinocyte differentiation and promotes proliferation through the cofilin/STAT3/Myc axis. If C-Raf is ablated, Ras-induced epidermal tumors do not form, and established ones regress [86]. In endothelial cells, C-Raf is needed to translocate Rok-α to VE-cadherin-based cell junctions. Here, Rok-α signaling stabilizes nascent adherens junctions, allowing the collective migration of endothelial cells required for sprouting angiogenesis [87]. In fibroblasts and embryonic liver, inhibition of Rok-α by C-Raf controls the trafficking of the death receptor Fas, counteracting apoptosis by setting the threshold of Fas sensitivity [88].

Finally, a Mek-independent function of C-Raf was also demonstrated in K-Ras-driven lung cancer models, but its molecular basis is currently unclear [89,90].

Similar to C-Raf, A-Raf has been reported to regulate Mst2 independently of its kinase activity [91]. B-Raf-dependent cross-talk with other pathways has not been reported so far, but it is conceivable that, by binding to C-Raf, B-Raf may influence its interaction with other proteins. Indeed, C-Raf:Rok-α complexes are more abundant in B-Raf ablated keratinocytes [92], a fact that could possibly affect the intensity of the cross-talks mentioned above.

## 6 Raf and Mek inhibitors – success stories with pitfalls

Activating B-RafV600E kinase mutations occur in up to 60% of melanomas (http://www.cbioportal.org), making B-Raf an attractive drug target for this malignancy. In the last years, significant efforts have been made to find compounds that inhibit B-RafV600E and, ideally, cause melanoma regression.

New generation ATP-competitive *Raf inhibitors*, such as the clinically approved vemurafenib (PLX4032) [93–97] and dabrafenib (GSK2118436) [96,98], have improved selectivity for mutant B-RafV600E (Table 1), resulting in high response rates and increased progression-free and overall survival in patients with BRAF mutant melanoma. However, cutaneous toxicities, such as the onset of squamous cell carcinomas and keratoacanthomas, occur in melanoma patients treated with vemurafenib (30%) or dabrafenib (7%) [93,99]. These lesions contain activated Erk, a paradoxical finding considering that the patients are treated with an inhibitor of the pathway.

Several explanations can be put forward to rationalize the Raf inhibitor paradox. The first involves Raf heterodimerization. In the presence of active Ras and of limited amounts of the inhibitor, Raf kinases adopt a conformation promoting hetero- and homodimerization. In the context of these dimers, the drug-bound protomer transactivates the inhibitor-free Raf kinase [107,108]. A more complex mechanism, suggesting relocalization of B-Raf from an inhibitory cytosolic complex and allowing RAS:B-Raf:C-Raf complex formation in Ras-transformed cells, has also been proposed [24].

What is common to these models is that they both rely on Ras activation as an initial event for inhibitor-induced Erk activation and tumorigenesis. Ras mutations were found in secondary skin lesions of melanoma patients treated with Raf inhibitors [109,110], and the significance of Ras activation for Raf inhibitor-induced tumor development was verified in mouse models in which Ras activation was induced either by chemical carcinogenesis [110] or by targeting the GEF SOS to the plasma membrane of basal keratinocytes [92]. The latter model confirmed that Ras activation, in the absence of other mutations or of inflammation, is enough to trigger Raf inhibitor-driven tumorigenesis in both skin and gastric epithelia [92]. Thus, Ras + inhibitor-induced Erk activation accelerates the growth of tumors originating from cells containing activated Ras [92,109,110]. Further common to both models is the concept that paradoxical activation of the pathway can only happen at low inhibitor concentration [92,107,108,110]. Unfortunately, the use of saturating concentrations of the inhibitor is precluded by high cytotoxicity [100].

In mouse models, B-Raf and C-Raf are required for Ras + vemurafenib driven tumorigenesis. However, only C-Raf is necessary for tumor development induced by GDC-0879, another ATP-competitive Raf inhibitor [111]. This specific role of C-Raf is not mediated through Erk phosphorylation, which is similar in B- or C-Raf-deficient epidermis and is necessary, but not sufficient, for the development of inhibitor + Ras induced tumors. Concomitant dedifferentiation, induced by C-Raf as endogenous inhibitor of the Rok-α signaling pathway [85,86,112], is indispensable for tumor development [92]. Rok-α inhibition by C-Raf-Rok-α interaction is not affected by Raf-inhibitor-induced Raf hetero- or homodimerization, but in general more C-Raf binds to Rok-α in B-Raf-deficient K5-SOS-F epidermis. The basis of this finding is currently unknown, but it is possible that B-Raf ablation frees up an additional pool of C-Raf for Rok-α interaction. The fact that Ras + Raf-inhibitor–-induced tumorigenesis requires both Raf-mediated Erk activation and kinase-independent Rok-α inhibition by C-Raf suggests that combination therapies targeting kinase and non-kinase functions of Raf may be more efficient and safer for the treatment of skin tumors.

More recently, a Ras-independent mechanism by which Raf inhibitors can activate the Erk pathway has been described. Raf inhibitors have been shown to block the autoinhibitory P-loop phosphorylation that regulates wild-type Raf, but not BRafV600E. This mechanism brings about the activation of wild-type Raf monomers, is independent of Ras binding and of inhibitor-induced dimerization, and may potentially have more widespread consequences than the mechanisms relying on activated Ras [113].

In addition to the onset of side effects due to paradox pathway activation, the therapeutic success of Raf inhibitors is compromised by acquired drug resistance. Several mechanisms have been described by which cells can evade Raf inhibition. A prominent one is pathway reactivation by upstream components, such as receptor tyrosine kinases (RTKs; EGFR, PDGFRβ, IGF-1R, MET through HGF secretion by the tumor microenvironment) [114–118]. As a specific example, in BRafV600E-expressing melanoma cells Raf inhibitors disable the Erk dependent feedback which suppresses RTK-Ras signaling, reactivating mitogenic signaling [119]. Resistance caused by RTK reactivation is not restricted to melanoma, being observed in B-RafV600E expressing colon carcinoma cells [120,121]. This mechanism has the additional “advantage” of enlisting parallel survival pathways, such as the PI3K/AKT cascade [116–118,122], whose activation strongly reduces the sensitivity of K-Ras mutant cancer to Erk inhibition [123].

Drug resistance can also be engendered in B-Raf-mutant tumors by direct pathway reactivation, caused by secondary N-Ras [115] and Mek1 mutations [124,125] or by target amplification/diversification. B-RafV600E amplification [126], expression of B-RafV600E splice variants promoting Ras-independent dimerization (p61B-RafV600E) [30], C-Raf overexpression [116,122,127], Raf isoform signal switching [116] and increased expression of the alternative Mek kinase COT (Tpl2) [128] have all been reported and connected to inhibitor resistance in melanoma cells. Finally, a gain of function resistance study revealed that a melanocyte-specific signaling circuit involving the transcription factors CREB and MITF is also able to mediate drug resistance [129].

Unlike Raf inhibitors, *Mek inhibitors* are unfortunately rather toxic for normal tissues, which currently limits their clinical use [130]. However, since the spectrum of activity of Mek inhibitors is predicted to be broader, many efforts are being carried out to develop more efficacious, less toxic substances, and to better understand their mechanism of action.

In this context, one puzzling finding has been that some allosteric Mek inhibitors suppress Erk signaling and proliferation less effectively in K-Ras-driven than B-RafV600E-driven tumors [131,132]. This conundrum has been resolved recently by the Rosen and the Hatzivassiliou groups. Using inhibitors that preferentially bind to active or inactive Mek, they have shown that the first class of substances is more effective in cells harboring B-RafV600E, which have high concentrations of phosphorylated Mek, while the second is more effective in K-Ras transformed cells, where the concentration of phosphorylated Mek is lower [133]. This is due to the fact that in Ras transformed cells, signaling through the Erk pathway is reduced by the phosphorylation of negative regulatory residues of C-Raf by Erk, which inhibits both C-Raf kinase activity and its interaction with Ras. Mek inhibitors interfere with this negative feedback loop, and the resulting C-Raf reactivation limits their efficacy in this system [132]. B-Raf mutant tumors are insensitive to this negative feedback [134], which explains why Mek inhibitors can more effectively downregulate the Erk pathway in these cells.

The second class of inhibitors interferes with the binding of Raf to Mek, improving efficacy in K-Ras transformed cells. Within this class, inhibitors such as Selumetinib, PD0325901, and CH5126766 (Table 1), stabilize a complex in which Mek cannot be phosphorylated by Raf, essentially generating a dominant-negative inhibitor of Raf [106,132]. Stabilization of the Raf:Mek complex also has negative consequences for the formation of Raf heterodimers, and therefore for Ras-induced Raf activation [133].

In contrast, the allosteric inhibitor Trametinib reduces Raf binding to Mek. Trametinib inhibits the proliferation of both Ras and B-RafV600E mutant cell lines and xenografts [132,135] and decreases both Ras and Ras + Raf inhibitor-induced tumor formation in a transgenic model of Ras-driven epidermal tumorigenesis [92]. Trametinib is also the only Mek inhibitor that has been approved as a single agent for treatment of unresectable or metastatic melanoma harboring the B-Raf V600E or V600K mutation.

From all of the above, it is clear that alternative therapeutic strategies are needed to overcome drug resistance. Two main avenues are being explored. The first, approved for clinical use by the FDA on January 2014 for the treatment of metastatic B-Raf-driven melanoma, is a combination of Raf and Mek inhibitors (dabrafenib plus trametinib) [136]. This double hit is expected to circumvent and/or delay acquired resistance originating from pathway reactivation, and has been recently shown to prevent melanoma metastasis in a preclinical model [137]. It is likely that in the future, Raf inhibitor monotherapy will be replaced by Raf and Mek inhibitor combination therapy as the first-line treatment for B-Raf-driven melanoma.

In addition to this “vertical” inhibitor combination, preclinical studies have also shown the benefits of co-targeting PI3K, mTOR, Hsp90, CDK 4/6, FGFR, c-Met (“parallel” inhibitor combination) or using immunotherapy to overcome drug resistance (reviewed in [138,139]).

“Drug holidays”, the temporary cessation of drug treatment, may prove effective in reverting drug resistance. Raf inhibitor-resistant melanomas revert to drug-sensitivity when the treatment is interrupted in preclinical models [140] and, most importantly, in the clinic [141]. This indicates that drug resistance is adaptive and, most importantly, reversible. The preclinical model has shown that B-Raf-driven tumors become not only resistant, but addicted to Raf inhibitors in vivo, resulting in lethal, drug-resistant disease the onset of which can be delayed by intermittent inhibitor treatment [140]. Along the same lines, a recent report from the Bernards lab has shown that melanoma cells that survive Raf inhibitor treatment through the reversible upregulation of RTKs enter senescence due to supraphysiological Erk pathway stimulation when the drug is removed. These cells, which acquire inhibitor resistance at the cost of their general fitness, are negatively selected during “drug holidays”, restoring the sensitivity of the tumor population to the Raf inhibitor axis [114].

The last 5 years have seen tremendous progress in the area of clinical Erk pathway inhibition, with exceptional fast rates of bench-to-bedside translation. In addition, however, research on the inhibitors’ mode of action and on the mechanisms underlying resistance has advanced our understanding of how the Erk pathway is wired not only in tumors, but also in normal cells. Thus, this area of research represents a prime example of what can be achieved by the concerted efforts of academia, companies and clinicians investigating the same problems from different angles.

## 7 Conclusions

The investigation of the Ras/Raf/Mek/Erk pathway has provided us with a wealth of insight into the regulation of complex signaling networks, some of which are likely applicable to other MAPK cascades. Protein–protein interaction has emerged as one recurrent theme, with major consequences for pathway activation, regulation and cross-talk; it is becoming clear that using inhibitors that target complex formation in addition to catalytic activity may yield superior specificity and broaden the range of susceptible tumors. The dynamics of complex formation strongly depend on the stoichiometry of the proteins involved in the interaction(s), which in turn varies in different healthy tissues and is individually modulated in transformed cells. The resulting changes in the assembly and localization of signaling complexes are likely to specify individual biochemical and biological outcomes. The big challenge now will be to obtain a complete map of the interaction partners in different tissues, to determine which of the interactions are essential in development and disease, and identify those that can be exploited for the purpose of molecule-targeted therapy.

## Figures and Tables

**Table 1:** FDA-approved Raf and Mek inhibitors.

	Target IC_50_ (nM)				
	B-Raf	B-Raf^V600E^	C-Raf	Mek1	Mek2
Raf inhibitors					
Vemurafenib⁎ (PLX4032 [100])	100	31	48	n.a.	n.a.
Dabrafenib⁎ (GSK2118436 [101])	3.2	0.80	5	n.a.	n.a.
GDC-0879 [102]	n.a.	0.13	n.a.	n.a.	n.a.

Mek inhibitors					
Trametinib⁎ (GSK1120212 [103])	n.a.	n.a.	n.a.	0.92	1.80
Selumetinib (AZD6244 [104])	n.a.	n.a.	n.a.	14	n.a.
PD0325901 [105]	n.a.	n.a.	n.a.	0.33	0.33
CH5126766 [106])	19.0	8.20	56	160	n.a.

⁎ Vemurafenib, dabrafenib and trametinib have been approved by the FDA as single agent therapy for the treatment of unresectable metastatic melanomas harboring the B-Raf^V600E^ mutation; dabrafenib and trametinib have also been approved by the FDA as combination therapy for the same disease. n.a., not applicable.
